# 15-Deoxy-∆-^12,14^-Prostaglandin J2 (15d-PGJ2), an Endogenous Ligand of PPAR-*γ*: Function and Mechanism

**DOI:** 10.1155/2019/7242030

**Published:** 2019-08-01

**Authors:** Jingjing Li, Chuanyong Guo, Jianye Wu

**Affiliations:** ^1^Department of Gastroenterology, Putuo People's Hospital, Tongji University School of Medicine, Shanghai 200060, China; ^2^Department of Gastroenterology, Shanghai Tenth People's Hospital, Tongji University School of Medicine, Shanghai 200072, China

## Abstract

15-Deoxy-∆-^12,14^-prostaglandin J2 (15d-PGJ2), a natural peroxisome proliferator-activated receptor-*γ* (PPAR-*γ*) agonist, has been explored in some detail over the last 20 years. By triggering the PPAR-*γ* signalling pathway, it plays many roles and exerts antitumour, anti-inflammatory, antioxidation, antifibrosis, and antiangiogenesis effects. Although many synthetic PPAR-*γ* receptor agonists have been developed, as an endogenous product of PPAR-*γ* receptors, 15d-PGJ2 has beneficial characteristics including rapid expression and the ability to contribute to a natural defence mechanism. In this review, we discuss the latest advances in our knowledge of the biological role of 15d-PGJ2 mediated through PPAR-*γ*. It is important to understand its structure, synthesis, and functional mechanisms to develop preventive agents and limit the progression of associated diseases.

## 1. Introduction

Prostaglandins are lipid signalling molecules with multiple functions that are produced from arachidonic acid by cyclooxygenase [1]. Most prostaglandins activate a variety of intracellular signalling pathways and stimulate various biological activities by activating specific G protein-coupled receptors on cell membranes [2, 3]. Most prostaglandins exert proinflammatory effects, but cyclopentenone prostaglandins reportedly exert anti-inflammatory effects. 15-Deoxy-∆-^12,14^-prostaglandin J2 (15d-PGJ2), the most widely studied cyclopentenone prostaglandin, was also the first endogenous ligand of peroxisome proliferator-activated receptor-*γ* (PPAR-*γ*) to be identified [4]. However, PPAR-*γ* plays an important role in lipid and carbohydrate metabolism, inflammation, and the proliferation and differentiation of many cell types in different tissues [5, 6]. The present review summarises the health benefits of 15d-PGJ2 related to various diseases and the PPAR-*γ* signalling pathway.

### 1.1. Structure of 15d-PGJ2

**Figure 1:**
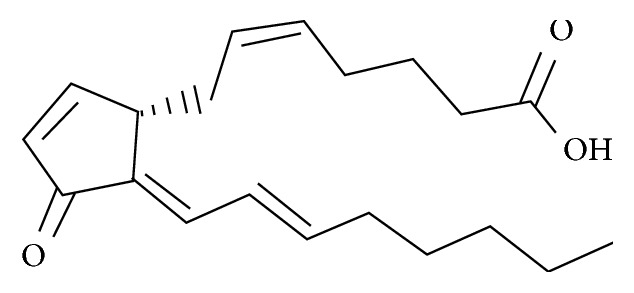
Structure of 15d-PGJ2.

15d-PGJ2 is a prostaglandin derived from arachidonic acid. It is an unsaturated carboxylic acid composed of a 20-carbon skeleton that includes a five-membered ring. It has highly active alpha- and beta-unsaturated carbonyl groups that can covalently bind to mercaptan groups of various proteins, thereby altering their functions. 15d-PGJ2 is a cyclopentenone prostaglandin with broad biologically activity that generally exists in liquid form (Figure 1). The molecular formula of 15d-PGJ2 is C20H28O3, and the molecular mass is 316.4 kDa.

### 1.2. Biosynthesis of 15d-PGJ2

**Figure 2:**
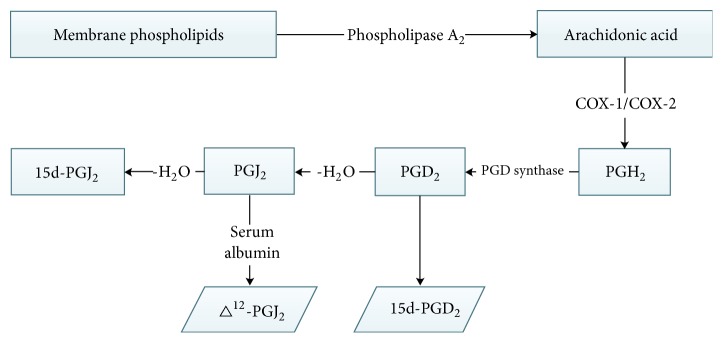
Biosynthesis of 15d-PGJ2.

15d-PGJ2 is the most widely studied metabolite of the prostatic family of PGJ2 compounds. It can react rapidly with important cell nucleophiles such as cysteine sulfhydryl groups of proteins via the MELK addition reaction, thereby altering biological activity [7]. First, arachidonic acid in membrane phospholipids is induced by phospholipase A, and unstable endoperoxide prostaglandin H2 (PGH2) is produced by cyclooxygenase 1 and 2 (COX-1 and -2). In the presence of sulfhydryl complexes, PGD2 synthase catalyses the isomerisation of PGH2 to PDGD2, PGE2, PGF2alpha, PGI2, and thromboxane A2 that interact with specific receptors. PGD2 can spontaneously release water molecules to form PGJ2, partly dependent on serum albumin, resulting in ∆12PGJ2 and other molecules, and 13,14 double bond rearrangement and dehydration yield 15d-PGJ2 (Figure 2).

### 1.3. 15d-PGJ2 Regulation of PPAR-*γ*

**Figure 3:**
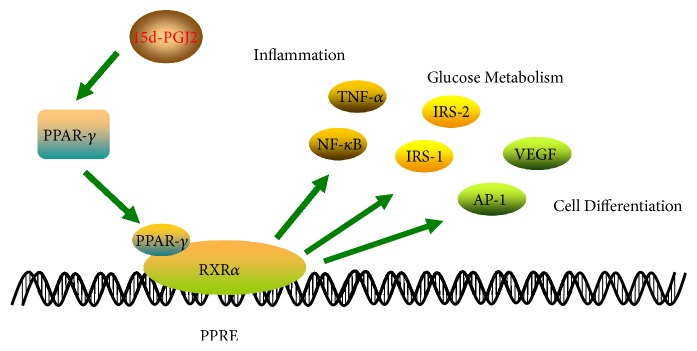
Regulation of PPAR-*γ* by 15d-PGJ2.

The N-terminal functional region of PPAR-*γ* contains a phosphorylation site mitogen-activated protein kinase (MAPK). After 15d-PGJ2 enters cells and binds to PPAR-*γ*, activation results in the formation of heterodimers with retinoid X receptor alpha (RXR*α*) and subsequent binding to specific DNA sequences to activate expression of target genes [8]. This specific DNA sequence, found in genes encoding hexanoyl coenzyme A synthase, lipoprotein lipase (LPL), insulin receptor substrate-2 (IRS-2), leptins, and tumour necrosis factor-alpha (TNF-*α*), is known as the peroxisome proliferator response element (PPRE) [9]. Simultaneously, PPAR can also affect nuclear factor-kappa B (NF-*κ*B), activator protein-1 (AP-1), and JAK/STAT, which further regulates the expression of downstream related genes and plays an important role in fat formation, glycolipid metabolism, inflammatory responses and immunity (Figure 3) [10–13].

## 2. Bioavailability of 15d-PGJ2 Related to PPAR-*γ*

PPAR-*γ* is the main target of many natural compounds and is closely related to cancer, inflammation, hypertension, type 2 diabetes, and other diseases [14–16]. 15d-PGJ2 is one of the most well defined PPAR-*γ* ligands, and its anticancer and anti-inflammatory effects may or may not be dependent on PPAR-*γ*. This review summarises recent findings regarding the functions and mechanisms of 15d-PGJ2 related to PPAR-*γ*.

### 2.1. Antitumour Activity

Studies have shown that the natural PPAR-*γ* agonist 15d-PGJ2 exerts anticancer activity by promoting apoptosis, resisting angiogenesis, and inhibiting migration and stem cell activity [6]. As a PPAR-*γ* ligand, 15d-PGJ2 can induce terminal differentiation and inhibit the growth of lung and colon cancer cells by inhibiting DNA synthesis [17, 18]. Its functions can be divided into PPAR-*γ*-dependent and semi-dependent types. One group suggested that 15d-PGJ2 can regulate the myc/mad/max network via PPAR-*γ* to promote cell apoptosis by inhibiting the expression of human telomerase reverse transcriptase (hTERT) and telomerase activity in colon cancer cells [19]. Another group demonstrated that 15d-PGJ2 may play an anticancer role in gastric cancer [20] and oral squamous cell carcinoma cells [21] by promoting cell apoptosis. Although 15d-PGJ2 is an endogenous ligand of PPAR-*γ*, it promotes apoptosis of cancer cells, and this is not entirely dependent on PPAR-*γ*. Han and colleagues found that 15d-PGJ2 enhanced TRAIL-induced apoptosis by downregulating AKT expression and phosphorylation. The sensitivity of 15d-PGJ2 to TRAIL-induced apoptosis was not completely blocked by PPAR-*γ* inhibitor GW9662, suggesting that 15d-PGJ2 is not completely dependent on PPAR-*γ* [22]. In addition, 15d-PGJ2 sensitises cancer cells to TNF-like weak activators through a reactive oxygen species (ROS-)dependent cell death pathway and may have chemotherapeutic effects as an apoptotic enhancer [23]. Consistent with this proposal, Fulzele and colleagues (2007) confirmed that the mechanisms of 15d-PGJ2 (combined with docetaxel) on apoptotic induction in lung cancer are both PPAR-*γ*-dependent and -independent [24]. These results suggest that PPAR-*γ* activation may be a key factor in inducing apoptosis of cancer cells. Therefore, 15d-PGJ2 may promote apoptosis of cancer cells in a PPAR-*γ*-dependent manner, and the PPAR-*γ* ligand may be a new anticancer agent worthy of further study.

Neovascularisation is a key mechanism of tumorigenesis, development, and rapid metastasis. Therefore, inhibiting angiogenesis is a crucial factor in cancer treatment. It has been reported that 15d-PGJ2 has antineovascularisation effects. Fu and Yuan found that angiogenesis is inhibited by 15d-PGJ2 via downregulation of angiopoietin-1 [25] and vascular endothelial growth factor [26] in gastric and renal cancer. 15d-PGJ2 inhibits overexpression of COX-2 and alters the expression of important angiogenesis regulators in various human malignant tumours [27].

The specificity of 15d-PGJ2 for proliferation and invasion of cancer cells also plays an important role in cancer therapy [28]. The PPAR-*γ* ligand 15d-PGJ2 can inhibit the growth of oesophageal adenocarcinoma cells by inducing cell cycle arrest, combined with promoting apoptosis and reducing ornithine decarboxylase activity [29]. Furthermore, cell cycle arrest at the G2/M phase and apoptosis of human endothelial cells induced by 15d-PGJ2 result in growth arrest of a uterine cancer cell line [30]. Another study showed that 15d-PGJ2 is a microtubule protein binding agent that can disrupt the stability of microtubules and induce mitotic arrest, leading to breast cancer cell death [31]. In terms of migration and invasion, 15d-PGJ2 is believed to reduce the expression of matrix metalloproteinase (MMP)-2 and MMP-9, thereby inhibiting the invasiveness of breast cancer [32] and pancreatic cancer cells [33, 34]. In addition, 15d-PGJ2 inhibits the proliferation and invasiveness of colon cancer cell lines via a mechanism related to G1 cell cycle arrest, and downregulation of MMP-7 synthesis [35] and CXCR4 via PPAR-*γ* and NF-kappa B [10]. Together, these studies have shown that 15d-PGJ2 can simultaneously promote apoptosis and inhibit migration, which can comprehensively inhibit tumour progression.

Recent studies have shown that cancer stem cells play an important role in the initiation and maintenance of tumour growth, and 15d-PGJ2 can control proliferation to a certain extent [36]. However, research on the effect of 15d-PGJ2 on the proliferation of cancer stem cells is in its infancy. First, PPAR-*γ* agonists have been identified as markers of the inhibition of growth and progression of brain cancer stem cells [37]. However, the effect of 15d-PGJ2 on cancer stem cells and its application to anticancer therapy require further exploration, as do potential therapeutic applications. The antineoplastic effects of 15d-PGJ2 are summarised in Table 1.

### 2.2. Anti-Inflammatory and Antioxidant Activity

PPAR-*γ* is expressed in human endothelial cells, vascular smooth muscle cells, and monocytes [38, 39]. 15d-PGJ2 can effectively regulate T-cell activation, expression of surface proteins, and related inflammatory cytokines by enhancing PPAR-*γ* transcriptional activity [40]. Similar to most other PGs, 15d-PGJ2 displays anti-inflammatory and antioxidative activities, e.g., via inhibition of NF-kappa B and JAK-STAT pathways [41, 42]. This section of the review focuses on the anti-inflammatory and antioxidative effects of 15d-PGJ2.

Activation of macrophages leads to the production of various proinflammatory mediators, such as IL-6, TNF-*α*, IL-1*β*, and inducible nitrate oxide synthase (iNOS), among which the activation of PPAR-*γ* plays a negative regulatory role [43, 44]. Meng and colleagues used the natural PPAR-*γ* ligand 15d-PGJ2 to stimulate mouse-derived RAW264.7 macrophage cell line and found that angiotensin-II-induced expression of EGR-1, ROS, and inflammatory factors (IL-1*β*, TNF-*α*, TGF-*β*, MCP-1, and ICAM-1) was significantly reduced, while macrophage migration and proliferation were inhibited [45]. In chronic liver injury, 15d-PGJ2 decreases the number and activation of bone marrow (BM)-derived macrophages (BMMs) in damaged liver tissue and inhibits the expression of inflammatory cytokines such as MIP-1*β*, TNF-*α*, and NOS2 [46]. Endothelial cells also express large quantities of PPAR-*γ*, and Marcone et al. (2016) suggested that 15d-PGJ2 may modify proteasomes in human endothelial cells and inhibit the NF-*κ*B inflammation-mediated pathway [47]. Similarly, 15d-PGJ2 can protect brain endothelial cells from apoptosis induced by hypoxia by inhibiting the transcription of p22phox [48]. In human retinal pigment epithelial cells, 15d-PGJ2 also inhibits lipopolysaccharide (LPS)-stimulated inflammation by enhancing the activity of platelet-activating factor acetyl hydrolase in conjunction with PPAR-*γ* [49].

The accumulated evidence suggests that, in animal models, 15d-PGJ2 plays an indispensable anti-inflammatory and antioxidative role. Firstly, 15d-PGJ2 can prevent concanavalin A-induced autoimmune hepatitis by reducing the release of proinflammatory cytokines, which is related to the activation of PPAR-*γ* and decreased NF-*κ*B activity [50]. This mechanism was validated in a HepG2 cell model* in vitro* [51]. It was also demonstrated that 15d-PGJ2 downregulates the activin receptor and Smad pathways [52]. In an endotoxin-induced lung injury model, 15d-PGJ2 has been shown to reduce the levels of TNF-*α* and ICAM-1 by inhibiting the activity of NF-*κ*B [53]. In other studies, 15d-PGJ2 protected rat lung tissue from gastric inhalation injury and reduced infection or allergy-induced pulmonary inflammation by inhibiting the production of proinflammatory cytokines (TNF-*α* and IL-10) [54, 55] and gene expression of chemokines (CCL2, CCL3, CCL4, and CXCL10) [56]. During protection of the nervous system, 15d-PGJ2 is important. M2 microglia can promote neurogenesis and oligogenesis of nerve stem/progenitor cells through the PPAR-*γ* signalling pathway [57, 58]. PPAR-*γ* agonists can control inflammation and protect neurons from degenerative diseases of the central nervous system such as Alzheimer's disease, Parkinson's disease, and multiple sclerosis by inhibiting activated microglia and via PPAR-*γ*. Huang et al. found that 15d-PGJ2, a recognised endogenous ligand of PPAR-*γ*, is increased in the supernatant of M2 phenotype cells. After cerebral perfusion of 15d-PGJ2, expression of TNF-*α* and IL-1*β* decreases, the proportion of apoptotic cells decreases, cerebral infarction and brain oedema decrease, and neurological deficits decrease, suggesting that it regulates the neuroprotective effects of microglia [59]. Similarly, pretreatment with 15d-PGJ2 also inhibits the activation of nitrogen oxides and ROS in a PPAR-*γ*-dependent manner [60], thereby alleviating neuronal damage [61]. Another viewpoint is that 15d-PGJ2 exerts neuroprotective effects by mediating neuronal autophagy following cerebral ischemia-reperfusion injury [62]. In addition, in gouty arthritis, 15d-PGJ2 inhibits inflammation by reducing the release and oxidative stress levels of IL-1*β*, TNF-*α*, IL-6, IL-17, and IL-33 [9]. 15d-PGJ2 can also inhibit the prostaglandin synthesis pathway in activated chondrocytes and regulate the anti-inflammatory circuit by regulating heat shock 70 (HSP70) to reduce the levels of NF-*κ*B, COX-2, and oxidative stress in chondrocytes [63]. Furthermore, 15d-PGJ2 can significantly reduce eosinophil production and migration in the abdominal cavity, via IL-23/IL-17 and IL-33, and exert therapeutic effects on eosinophil-induced diseases [64]. In acute pancreatitis, 15d-PGJ2 also attenuates the expression of TLR4 in acinar cells and inflammatory responses and reduces the severity of acute pancreatitis [65].

In summary, 15d-PGJ2 was the first endogenous ligand of PPAR-gamma to be discovered. It plays anti-inflammatory, antioxidative, and protective roles in brain injury induced by stress, acute pancreatitis, drug-induced lung injury, and ischemia-reperfusion in animal models of monocytes, endothelial cells, macrophages, and other inflammatory diseases. The anti-inflammatory and antioxidant effects of 15d-PGJ2 are summarised in Table 2.

### 2.3. Antifibrotic Activity

Organ fibrosis has always been a difficult problem for the scientific community to address, because it can lead to progressive dysfunction of various organs [66, 67]. The main pathological mechanism is excessive activation of TGF-*β*. Ligands of PPAR-*γ* may depend on activating PPAR-*γ* to block TGF-*β* signalling and inhibit tissue and organ fibrosis [68, 69]. Of course, some mechanisms may also play an antifibrotic role independent of PPAR-*γ* activation. Herein, we systematically review studies on the antifibrosis mechanism of 15d-PGJ2.

In studies on pulmonary fibrosis, 15d-PGJ2 was found to inhibit the differentiation of myofibroblasts driven by TGF-*β* and the production of type I collagen, and its effects are both dependent on and independent of PPAR-*γ* [70]. PPAR-*γ* activation leads to the transformation of hepatic stellate cells (HSCs) from an activated to a stationary state. 15d-PGJ2 can strongly inhibit the proliferation of HSCs, and the expression of connective tissue growth factor (CTGF) in HSCs induced by TGF-*β*1, and inhibition can be significantly (but not completely) eliminated by pretreatment with PPAR-*γ* inhibitor GW9662. This indicates that PPAR-*γ* mediates the inhibition [71]. In the process of renal fibrosis, many kinds of cells participate together, including mesangial cells and fibroblasts, renal tubular epithelial cells, monocytes and macrophages, and lymphocytes [72]. Guo et al. (2004) proposed that 15d-PGJ2 may inhibit the activation of AP-1 and MAPKs by inhibiting TGF-*β*1, and it may inhibit the expression of fibronectin in mouse mesangial cells, thereby acting via dual mechanisms both dependent on and independent of PPAR-*γ* activation [73]. Wang et al. demonstrated that 15d-PGJ2 could reverse the TGF-*β*1/Smads signalling pathway and inhibit the activation of renal fibroblasts, CTGF expression, and extracellular matrix (ECM) synthesis in rat renal interstitial fibroblasts (NRK/49F) [74]. Finally, 15d-PGJ2 can also prevent the loss of the epithelial phenotype induced by TGF-*β*1 by activating PPAR-*γ*, and it can inhibit oxidative stress. Interestingly, specific knockout of PPAR-*γ* cannot play an effective role 15 days later. This indicates that targeting of PPAR-*γ* plays an important role in maintaining normal epithelial phenotype and fighting fibrosis in renal tubular epithelial cells [75]. In addition, 15d-PGJ2 also plays an important role in many other systems. For example, 15d-PGJ2 blocks TGF-*β*1-induced elevation of CTGF in cat corneal fibroblasts [76]. In skin fibroblasts, PPAR-*γ* activation can also eliminate the stimulation of collagen gene expression induced by TGF-*β*1, as well as Smads-dependent promoter activity in myofibroblast differentiation and normal fibroblasts, revealing a new method for controlling scleroderma fibrosis [77]. Fu et al. (2001) also found that 15d-PGJ2 significantly inhibits CTGF production induced by TGF-*β*1 in human aortic smooth muscle cells in a dose-dependent manner, and activation of PPAR-*γ* was achieved by directly interfering with the Smad3 signalling pathway [78].

Due to the broad tissue distribution and complex functions of PPAR-*γ*, its agonists play important physiological roles. Strong antifibrosis effects caused by inhibiting the TGF-*β* signalling pathway are clearly important [79]. In conclusion, evidence suggests that TGF-*β* is a key mediator of fibrous tissue. As a PPAR-*γ* agonist, 15d-PGJ2 has a significant inhibitory effect on TGF-*β* signal transduction and is an effective antifibrosis drug. The antifibrotic effects of 15d-PGJ2 are summarised in Table 3.

### 2.4. Other Biological Activities

15d-PGJ2 also plays an important role in other diseases. During the development of osteoporosis, 15d-PGJ2 may inhibit the expression of osteoblast marker genes in bone marrow cells by activating PPAR-*γ* transcriptional activity, which may be one of the reasons for 15d-PGJ2 involvement in age-related osteoporosis [80]. During vascular remodelling, the PPAR-*γ* agonist 15d-PGJ2 inhibits Ang II-induced cell proliferation and expression of KLF5 and cyclin D1 in vascular smooth muscle cells with growth arrest in dose-dependent manner, which provides new evidence for the beneficial vascular effects of PPAR-*γ* activation [81]. Additionally, 15d-PGJ2 inhibits activation of signal transducers and STAT3 in neuronal cells (SH-SY5Y-Ob-Rb cells) induced by leptin through the PPAR-*γ* pathway [82].

## 3. Other Molecular Targets of 15d-PGJ2

The effect of 15d-PGJ2 can be achieved independently of PPAR-*γ*. For example, in cancer research, Shin et al. demonstrated that 15d-PGJ2 can induce apoptosis in leukaemia and colorectal cancer cells through inactivation of AKT mediated by reactive oxygen species, further verifying its antitumour activity in vivo [83]. Ho and his colleagues proposed that 15d-PGJ2 induces vascular endothelial cell apoptosis through JNK signalling and p38 MAPK-mediated p53 activation both in vitro and in vivo [84]. In addition, 15d-PGJ2 plays an antitumour role by upregulating death receptor 5 expression in HCT116 cells [85]. In the treatment of inflammation, 15d-PGJ2 can rapidly induce the transcription of cytokine signal transduction inhibitors (SOCS) 1 and 3 and inhibit the activity of JAK in activated glial cells, thereby performing an anti-inflammatory role [86]. Additionally, inflammatory factors such as IL-6, IL-8, and IFN-*γ* can be inhibited by 15d-PGJ2 through NF-*κ*B, NrF2, and JAK/STAT pathways rather than PPAR-*γ* [87–89]. Furthermore, 15d-PGJ2 plays a key role in the homeostasis of BMSCs via a mechanism dependent on ROS-induced damage of liver, but not PPAR-*γ*, which may represent a new strategy for the treatment of liver fibrosis [90]. 15d-PGJ2 inhibits the expression of chemokines in a PPAR-*γ*-independent manner, which is related to blocking the NF-*κ*B pathway. PPAR-*γ* agonists may therefore represent a key drug target for improving inflammation-related tubulointerstitial fibrosis [91]. In pulmonary fibrosis, 15d-PGJ2 regulates the extracellular signal-regulated kinase pathway by inhibiting the expression of TG2 rather than PPAR-*γ* [92]. Finally, 15d-PGJ2 can also play a potential role in lowering blood lipid by regulating the specific molecules of lipid metabolism, such as PPAR-*δ*, liver X receptor (LXR), farnesoid X receptor (FXR), and SIRT1. Its effect in different tissues may be related to the distribution of the above antibodies in tissues and the differences in affinity of 15d-PGJ2 [93–95].

## 4. Conclusion and Future Perspectives

15d-PGJ2 is a metabolic product of the PGJ2 prostate family. Two studies in 1995 showed that it can activate the transcription factor PPAR-*γ* [96, 97]. Although many new alternative drugs have been developed over the years, as an endogenous ligand, 15d-PGJ2 has advantages, including rapid expression. 15d-PGJ2 has been extensively explored in recent studies, which proves that it can prevent various harmful pathological changes* in vivo*, such as tumours, inflammation, oxidative stress, fibrosis, vascular remodelling, and lipid metabolism, and reveals its protective role related to PPAR-*γ* signalling pathways [4–6]. Herein, the structure, synthesis, and biological effects of 15d-PGJ2 are reviewed based on the latest literature, but there remain gaps in our knowledge. For example, 15d-PGJ2 produced* in vivo* is not sufficient to regulate most physiological processes, and external replenishment requires more stable carriers. Moreover, 15d-PGJ2 has a variety of pharmacological effects, many of which are antagonistic toward each other, as exemplified by the dual characteristics of inducing the synthesis of vascular endothelial growth factor and antiangiogenesis [71, 95]. Therefore, it is also very important to explore the conditions under which a certain pharmacological action takes place. Work is clearly needed to elucidate the biological effects of 15d-PGJ2 and related compounds in order to develop improved drug treatment regimens and therapies.

## Figures and Tables

**Table 1:** Anti-tumour activity of 15d-PGJ2.

Tumour type	Mechanism	Cell type	Dosage	References
Lung caner	Apoptosis	H841, A549, PC14	1–40 *μ*M	[17]
Colon caner	Apoptosis	HT-29	0–100 *μ*M	[18]
Colon caner	Apoptosis	Caco-2	10–45 *μ*M	[19]
Gastric cancer	Apoptosis, Cell cycle(G1)	MKN-7, MKN-28, MKN-45, AGS	0.1–10 *μ*M	[20]
Oral squamous cell Cancer	Apoptosis, Cell cycle(G2/M)	SCCa	10 *μ*M, 20 *μ*M	[21]
Lung caner	Drug combination	A549, H460, female athymic nu/nu mice	0–40 *μ*M 1 mg/kg/day intraperitoneal	[24]
Colon caner	Apoptosis	HT-29, Caco-2	10 *μ*M	[23]
Leukemic	Apoptosis	HL-60, K562, SNU-C4	0-20 *μ*M	[22]
Gastric cancer	Inhibition of Ang-1	MKN45, HUVEC	0–10 *μ*M	[25]
Renal caner	Apoptosis, Inhibition of VEGF	SMKT-R-1, R-2, R-3, and R-4,	0–50 *μ*M	[26]
Lymphoma	Inhibition of COX-2	U937, BAEC	0–10 *μ*M	[27]
Oesophageal cancer	Apoptosis, Cell Cycle(G1)	TE-7	0–10 *μ*M	[29]
Endometrial cancer	Apoptosis, Cell Cycle(G2/M)	HHUA, HEC-59	0–20 *μ*M	[30]
Breast caner	Apoptosis, Cell Cycle(G2/M)	MCF-7	0–10 *μ*M	[31]
Breast cancer	Invasion	MDA-MB-231	5 *μ*M	[32]
Pancreatic cancer	Invasion	AsPC-1, SUIT-2 BxPC-3, MIA PaCa-2, Panc-1	0–25 *μ*M	[33, 34]
Colon caner	Cell Cycle(G1), Invasion	SW480, LS174T	0–40 *μ*M	[35]
Colon caner	Invasion	HT-29	0–30 *μ*M	[10]
Brain tumour	Inhibition of stem cells	U87MG, T98G	0–10 *μ*M	[37]

**Table 2:** Anti-inflammatory and antioxidant activities of 15d-PGJ2.

Type	Mechanism	Cell type	Dosage	References
macrophage	IL-1*β*, TNF-*α*, TGF-*β*, MCP-1, ICAM-1	RAW264.7	5 *μ*M	[45]
macrophage	MIP-1*β*, TNF-*α*, NOS2	Primary liver macrophages	0.5–2.5 *μ*M	[46]
endothelial cells	NF-*κ*B, TNF-*α*, VCAM-1, ICAM-1	Aortic endothelial cells	10 *μ*M	[47]
endothelial cells	ROS, Apoptosis	Cerebral endothelial cells (CECs)	1 *μ*M	[48]
endothelial cells	IL-6, MCP-1, ICAM-1	Human ARPE19 retinal pigment epithelial cells	10–20 *μ*M	[49]
Liver	NF-*κ*B, TNF-*α*, IL-1*β*, IL-6	LO2 and RAW264.7 cells, mice	2 *μ*M, 30 *μ*g/mL	[50]
	COX-2	HepG2 cells	5 *μ*M, 10 *μ*M	[51]
	NF-*κ*B, IL-6, IL-8	HepG2 cells	2 *μ*M, 5 *μ*M	[52]
Lung	TNF-*α*, NF-*κ*B, ICAM-1, CINC-1	Rats	0.3 mg/kg	[53]
	TNF-*α*, CINC‐1, IL-10, NF-*κ*B	Mice	1 mg/kg	[54]
	IL-6, TNF-*α*, CCL2, CCL3, CCL4, CXCL10	Mice MDCK, Calu-3 cells	250 *μ*g/kg 10 *μ*M	[56]
Nervous system	TNF-*α*, IL-1*β*	Rats	200 *μ*g/kg/	[59]
	ROS	Primary neurons cells	5 *μ*M	[60]
	ROS, NOX	Primary cortical neurons	1 *μ*M	[61]
	Autophagy, ROS	Mice	10 *μ*L icv	[62]
Gout arthritis	IL-1*β*, TNF-*α*, IL-6, IL-17, IL-33, NF-*κ*B	Mice	3, 10, or 30 *μ*g/kg	[9]
Cartilage	IL-1*β*, NF-*κ*B, COX-2, HSP70	Chondrocytes	10 *μ*M	[63]
Chronic eosinophilia	IL-33, IL-17, IL-23	Mice	100, 300 or 1000 *μ*g/kg	[64]
Acute pancreatitis	TLR4, CCL2	Pancreatic acini cells	10 *μ*M	[65]

**Table 3:** Anti-fibrotic activity of 15d-PGJ2.

Organ	Mechanism	Cell type	Dosage	References
Lung	TGF-*β*1, *α*-SMA	Human lung fibroblasts	10 *μ*M	[70]
Liver	TGF-*β*1, CTGF	Rat hepatic stellate cells	1–20 *μ*M	[71]
Renal	TGF-*β*1, MAPKs, AP-1	murine mesangial cells (SV40 MES 13)	20 *μ*M	[73]
	TGF-*β*1, CTGF, *α*-SMA	rat renal interstitial fibroblasts (NRK/49F)	10 *μ*M	[74]
	TGF-*β*1, ROS	Human kidney-2 cells (HK-2)	10 *μ*M	[75]
Eye	TGF-*β*1, *α*-SMA	Cat corneal fibroblasts	5 *μ*M	[76]
Skin	TGF-*β*1, *α*-SMA, COL1A2	human dermal fibroblasts	10 *μ*M	[77]
Aorta	TGF-*β*1, CTGF	Human Aortic Smooth Muscle Cells	1–10 *μ*M	[78]
